# The Course of Circulating Small Extracellular Vesicles in Patients Undergoing Surgical Aortic Valve Replacement

**DOI:** 10.1155/2020/6381396

**Published:** 2020-04-18

**Authors:** Andreas Weber, Shining Sophie Liu, Letizia Cardone, Philipp Rellecke, Stephan Urs Sixt, Artur Lichtenberg, Payam Akhyari

**Affiliations:** ^1^Department of Heart Surgery, Medical Faculty, Heinrich-Heine-University, Düsseldorf, Germany; ^2^Department of Anaesthesiology, Medical Faculty, Heinrich-Heine-University, Düsseldorf, Germany

## Abstract

In the last years, increasing efforts have been devoted to investigating the role of small extracellular vesicles (sEVs) in cardiovascular diseases. These nano-sized particles (30-150 nm), secreted by different cell types, contain signalling molecules that enable participation in intercellular communication processes. In this study, we examined the course of circulating sEVs in patients undergoing surgical aortic valve replacement (SAVR) and correlated them with echocardiographic and standard blood parameters. Peripheral blood samples were collected from 135 patients undergoing SAVR preoperatively and at three follow-up points. Circulating sEVs were precipitated using Exoquick™ exosome isolation reagent and analyzed by nanoparticle tracking analysis (NTA). Our findings indicate that no more than 7 days after SAVR, there was a marked increase of circulating sEVs before returning to initial values after 3 months. Further, shear stress is not a trigger for the formation and release of circulating sEVs. Moreover, we pointed out a correlation between circulating sEVs and erythrocytes as well as LDH and creatinine levels in peripheral blood. Finally, all patients with a moderate prosthesis-patient mismatch as well as with an impaired left ventricular mass regression had lower levels of circulating sEVs 3 months after SAVR compared to their respective status before surgery. We conclude that in patients with aortic valve stenosis (AVS), sEVs may play an important part in mediating cell-cell communication and SAVR may have a crucial and lasting impact on their circulating levels. Besides, lower levels of sEVs portend to be associated with inferior recovery after major surgical interventions. The additional use of circulating sEVs beyond echocardiographic and laboratory parameters could have a prognostic value to estimate adverse outcomes in patients undergoing SAVR.

## 1. Introduction

Aortic valve stenosis (AVS), as the functional consequence of calcific aortic valve disease (CAVD), is the most common heart valve disease in the US and Europe and is the second most frequent cause for cardiac surgery [1, 2]. CAVD has been identified not only as a slow and progressive but also as an active and regulated process akin to atherosclerosis involving the creation of calcium nodules, lipoprotein accumulation, and chronic inflammation.

Circulating extracellular vesicles (EVs) are submicron membrane vesicles (<2 *μ*m) derived from platelets, red and white blood cells, endothelium, and some other cell types and are released into the extracellular environment [3, 4]. Originally believed to behave simply as inert cellular debris, cell-derived EVs are present in peripheral blood both in physiological and pathophysiological conditions in low concentrations [5, 6]. EVs represent a heterogeneous population and are generated from cell membranes by a number of mechanisms in response to cellular activations, cell injury, and apoptosis [5, 6].

In general, the release of circulating EVs can be triggered by several stimuli including shear stress, complement attacks, or membrane activation processes [7–9]. A distinction is made between vesicles released from the surface of plasma membranes, which expose membrane antigens representative of their cellular origin, termed microvesicles (MVs) or microparticles (MPs) and other circulating vesicles derived from intracellular multivesicular bodies fused with the plasma membrane, termed small extracellular vesicles (sEVs) or exosomes [10–12]. Circulating sEVs are specialized membranous nano-sized vesicles (30–150 nm) containing certain combinations of lipids, adhesion, and intercellular signaling molecules as well as other functional cytosolic components like miRNA and mRNA and play a pivotal role in regulating cell-cell communication [7, 13].

Elevated counts of circulating MPs have been documented in the pathogenesis of various disorders such as cancer, infectious diseases, and diabetes mellitus [14]. Further, an increasing number of studies highlight the diverse contribution of circulating vesicles, particularly MPs and sEVs, in the evolution of vascular diseases including atherosclerosis, neointima formation, and vascular repair, primary hypertension, pulmonary artery hypertension, and aortic aneurysm [11, 15–18]. In cardiovascular diseases, MPs could be identified as an important player in the pathogenesis as well as a biomarker of the active disease, which indicates their diagnostic importance [15, 16]. In patients with AVS, a distinct correlation between increased levels of MPs and higher transvalvular pressure gradients has been described, which suggests that formation and release of MPs may be shear stress dependent [17]. In contrast, sEVs present a largely unknown “cell-to-cell” communication system, which is now increasingly being investigated for diagnostic and therapeutic use in CVDs [16, 19, 20].

In the present work, we analyzed the course of circulating sEVs in patients undergoing surgical aortic valve replacement (SAVR) and correlated their circulating levels with echocardiographic and standard blood parameters to evaluate their potential as a prognostic as well as diagnostic tool.

## 2. Methods

### 2.1. Ethics Statement

The protocol of the cohort study was approved by the institutional ethical board of the University of Düsseldorf (Reference number: 3381) and conducted in accordance with the Declaration of Helsinki. All patients were of adult age and provided written informed consent to participate in this study.

### 2.2. Study Design and Patient Selection

**Figure 1:**
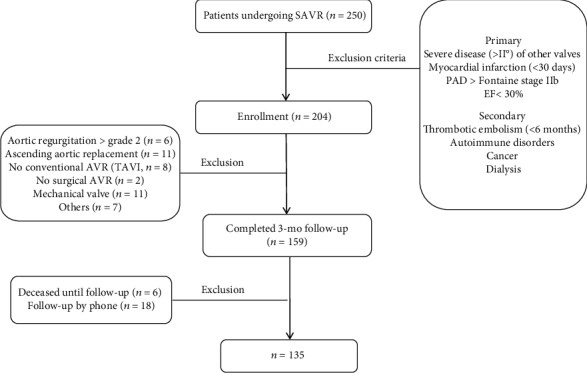
Flow diagram of the patient enrolment. SAVR: surgical aortic valve replacement; TAVI: transcatheter aortic valve implantation; PAD: peripheral artery disease; EF: ejection fraction.

Between July 2015 and September 2016, 250 consecutive patients undergoing cardiac surgery at the Department of Heart Surgery at the University Hospital Düsseldorf (UKD) were screened. Of these, 204 patients were identified to fulfill the inclusion criteria of moderate to severe AVS necessitating SAVR. Medical treatment and, in particular, all components of the surgical therapy including prosthesis choice were exclusively upon the discretion of the treating surgeon in accordance with the current recommendations and the patients' preferences. Patients with severe dysfunction (>II°) of other heart valves, myocardial infarction (<30 days), peripheral artery disease (>Fontaine stage IIb), reduced ejection fraction (<30%), thrombotic embolism (<6 months), autoimmune disorders, renal failure (requiring dialysis), and patients with previous cardiac surgery were excluded from further analysis (Figure 1). Moreover, in line with the secondary exclusion criteria, patients with concomitant aortic regurgitation (>II°), dilatation of the ascending aorta, or other indication for additional aortic surgery as well as patients receiving a mechanical valve were also excluded. Overall, a total of 8 patients, who were initially referred to SAVR, received a transcatheter aortic valve implantation (TAVI) and were therefore also excluded from further analysis.

### 2.3. Clinical Assessment and Data Collection

The following baseline data were collected: age, gender, weight and height, body mass index (BMI), New York Heart Association (NYHA) functional classification, degree of coronary artery disease (CAD), previous cardiac surgery, presence and severity of pulmonary hypertension, and other relevant comorbidities (i.e., chronic obstructive pulmonary disease (COPD), diabetes mellitus, dyslipidemia, arterial hypertension, peripheral vascular disease, cerebrovascular disease, and chronic kidney disease as evident by glomerular filtration rate (GFR)) as well as risk assessment scores (Euroscore II and STS PROM). Transthoracic echocardiography data were obtained with Doppler measurements prior to, 7 days (d) after and 3 months (mo) after SAVR. Echocardiographic studies were performed according to current recommendations by board certified physicians in the echo laboratory at the Department of Cardiovascular Surgery of UKD using current standard ultrasound systems (GE Vivid S5 or S6). AVS severity was graded according to current guidelines of the European Society of Cardiology (ESC). Left ventricular mass (LVM), LV-mass index (LVMI), and relative wall thickness (RWT) were calculated according to the recommendations of the American Society of Echocardiography [21]. Our local Central Research Institute for Clinical Chemistry and Laboratory Diagnostic determined the blood panel and analyzed various plasma markers (creatinine, creatinine kinase, high sensitive troponin T (hsTnT) and lactate dehydrogenase (LDH), C-reactive protein (CRP), and glutamate oxaloacetate transaminase (GOT)). For regular follow-up, patients were examined 7 d after SAVR before discharge from the hospital and once again invited to a 3 mo follow-up visit in the UKD study center. The follow-ups included medical history, physical examination, and transthoracic echocardiography. Prosthesis-patient mismatch (PPM) was defined as effective orifice area (EOA) indexed (EOAi) to body surface area (BSA) <0.85 cm^2^/BSA as moderate and <0.65 cm^2^/BSA as severe.

### 2.4. Isolation and Analysis of Circulating sEVs

Venous blood samples (5 mL) were collected from an antecubital vein into chilled BD vacutainer™ serum separation tubes (Vacutainer, Becton, Dickinson and Company, Franklin Lakes, New Jersey) at four points of time (preoperative (pre-OP), 24 h postoperative (post-OP), 7 d post-OP, and 3 mo post-OP). After 30 min clotting time, separation of serum was performed immediately by centrifuging at 1,700 x g for 15 minutes in a refrigerated centrifuge. Platelets were removed by centrifuging the serum samples at 3,000 x g at 4°C for 15 min. Circulating sEVs were precipitated from 250 *μ*L platelet poor serum using the Exoquick™ exosome isolation reagent (SBI, Palo Alto, CA, USA) according to manufacturer's instruction and resuspended in 30 *μ*L phosphate-buffered saline (PBS). Subsequently, samples were diluted 2.5∗10^5^-fold with ultrapure water and analyzed by nanoparticle tracking analysis (NTA, Zeta View, Particle Metrix, Meerbusch, Germany) as described previously [22, 23]. In preparation for this study, we validated the optimum parameters for NTA, so that the analysis of all samples could be conducted with identical acquisition parameters (supplementary table S1, S1 Fig).

### 2.5. Statistical Analysis

Results are expressed as median with interquartile range (IQR), mean with standard deviation (SD), or in percentage when appropriate. Echocardiographic and laboratory parameters were compared with the use of weighted Student's *t* test. Serum levels of sEVs were compared by using ordinary one-way ANOVA and Tukey's multiple comparisons test with single pooled variance. Linear regression and statistical analysis was performed by using GraphPad Prism 6. Significance levels are expressed as *p* < 0.05, *p* < 0.01, *p* < 0.001, and *p* < 0.0001.

## 3. Results

### 3.1. Characteristics of Study Population

A total of *n* = 159 patients receiving bioprosthetic AVs completed the 3 mo follow-up (Figure 1). In sum, 85 patients (53%) underwent isolated SAVR, while 74 patients (47%) received SAVR combined with coronary artery bypass grafting (CABG). Six patients deceased in the time up to the 3 mo follow-up; eighteen patients were followed up by phone or by contacting their general practitioners. These patients were excluded from further analysis.  Table 1 lists the demographic characteristics and medical history of the study patients. The mean age was 73.3 years (±7.1) and 82 (61%) patients were male. The most frequent comorbidity was hypertension, followed by dyslipidemia, diabetes mellitus type 2, and cardiac arrhythmia. Kidney function (GFR < 60 mL/min) was reduced in 37 (28%) patients. Bicuspid aortic valve occurred in 18 (13%) patients.

### 3.2. Echocardiographic Parameters

The echocardiographic parameters are illustrated in Table 2. As expected, peak gradient, mean gradient, peak jet velocity, and shear stress (peak jet velocity/LV-ejection fraction) were significantly diminished 7 d post-OP and at the 3 mo follow-up compared to pre-OP values. In parallel, EOAi values were remarkably increased (*p* < 0.0001). There was no significant change in left ventricular end-diastolic diameter (LVEDd), whereas left ventricular end-systolic diameter (LVESd) was decreased at the 3 mo follow-up (*p* = 0.0194). Intraventricular septal end-diastolic diameter (IVSd), posterior wall diameters (PWd), and anterior wall diameter (AWd) were reduced 1-week post-OP (IVSd: *p* = 0.0374; PWd: *p* = 0.0049; AWd: *p* = 0.0177) and at the 3 mo follow-up compared to pre-OP values (*p* < 0.0001). LVM and LVMI were remarkably reduced 7 d post-OP (LVM: *p* = 0.0027; LVMI: *p* = 0.0006) and at the 3 mo follow-up compared to pre-OP values (LVM: *p* = 0.0006; LVMI: *p* = 0.0002). RWT was significantly decreased at the 3 mo follow-up (*p* = 0.0003).

### 3.3. Laboratory Parameters

Laboratory parameters measured at the predefined time points are depicted in Table 3. There was no significant change in the thrombocyte levels, whereas leucocytes were significantly increased 7 d post-OP (*p* < 0.0001). Hemoglobin (Hb) and hematocrit (Hct) were remarkably reduced 7 d post-OP (*p* < 0.0001) and 3 mo post-OP (Hb: *p* = 0.0002; Hct: *p* = 0.0488). Creatinine kinase was significantly decreased 7 d post-OP (*p* < 0.0001) and 3 mo post-OP (*p* = 0.0305). CRP, hsTnT, LDH, and GOT were significantly increased 7 d post-OP (*p* < 0.001), whereas urea was remarkably increased 3 mo post-OP (*p* = 0.0124).

### 3.4. Course of Circulating sEVs

**Figure 2:**
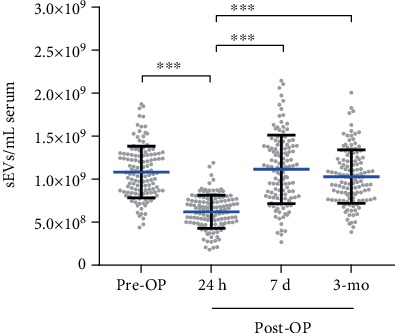
Serum levels of circulating sEVs. Levels of sEVs of 135 patients receiving SAVR at four points (pre-OP, 24 h post-OP, 7 d post-OP, and 3 mo post-OP). Mean (blue line) ± SD; ^∗∗∗^*p* < 0.001.

The mean levels of sEVs decreased significantly 24 h post-OP (*p* < 0.001), with a marked recovery thereafter at 7 d post-OP (Figure 2, S1 Fig, *p* < 0.001). At the 3 mo follow-up, the mean levels of sEVs for the entire study population equalized to initial values, i.e., pre-OP values. For further analysis, patients were divided into two groups based on their surgical procedure (S2 Fig). There were no significant differences in patients receiving isolated SAVR (*n* = 78) compared to patients undergoing SAVR combined with CABG (*n* = 57) at any points of time.

### 3.5. Correlation of Circulating sEVs with Demographic Parameters and Body Mass Index

**Figure 3:**
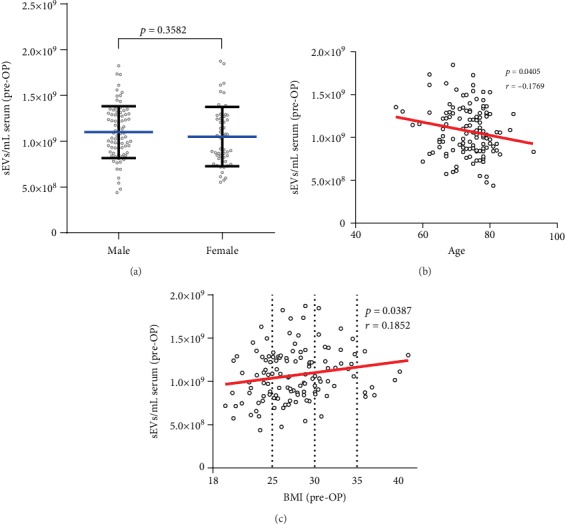
Correlation of pre-OP levels of sEVs with demographic parameters and BMI. (a) Gender-related differences of pre-OP levels of sEVs (mean ± SD) of 135 patients receiving SAVR. Correlation of pre-OP levels of sEVs with age (b) and BMI (c).

The pre-OP levels of sEVs displayed no gender-related differences (Figure 3(a), *p* = 0.3582), but demonstrated a significant negative correlation with age (Figure 3(b), *p* = 0.4051, *r*^2^ = 0.031) and a significant positive correlation with the BMI of the patients (Figure 3(c), *p* = 0.0387, *r*^2^ = 0.034).

### 3.6. Correlation of Circulating sEVs with Echocardiographic Parameters

**Figure 4:**
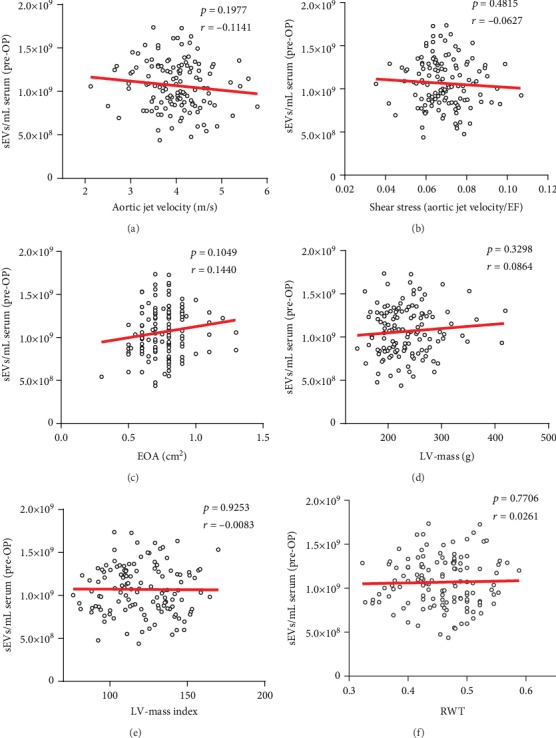
Correlation of sEVs with echocardiographic parameters in patients with AVS prior to SAVR. Linear regression of sEVs with aortic jet velocity (a), shear stress (b), effective orifice area (c), LV-mass (d), LV-mass index (e), and relative wall thickness (f) in patients before undergoing SAVR (*n* = 135).

There was no significant correlation between the pre-OP levels of sEVs with aortic jet velocity (Figure 4(a), *p* = 0.1977) or shear stress (Figure 4(b), *p* = 0.4815), but a positive trend with the EOA (Figure 4(c), *p* = 0.1049). Further, no correlation could be detected between pre-OP levels of sEVs and LVM, LVMI, and RWT (Figures 4(d)–(f)). Furthermore, no significant correlation between the levels of sEVs and echocardiographic parameters could be detected 7 d post-OP (S3 Fig) and at follow-up 3 mo post-OP (S4 Fig).

### 3.7. Correlation of Circulating sEVs with Laboratory Parameters

**Figure 5:**
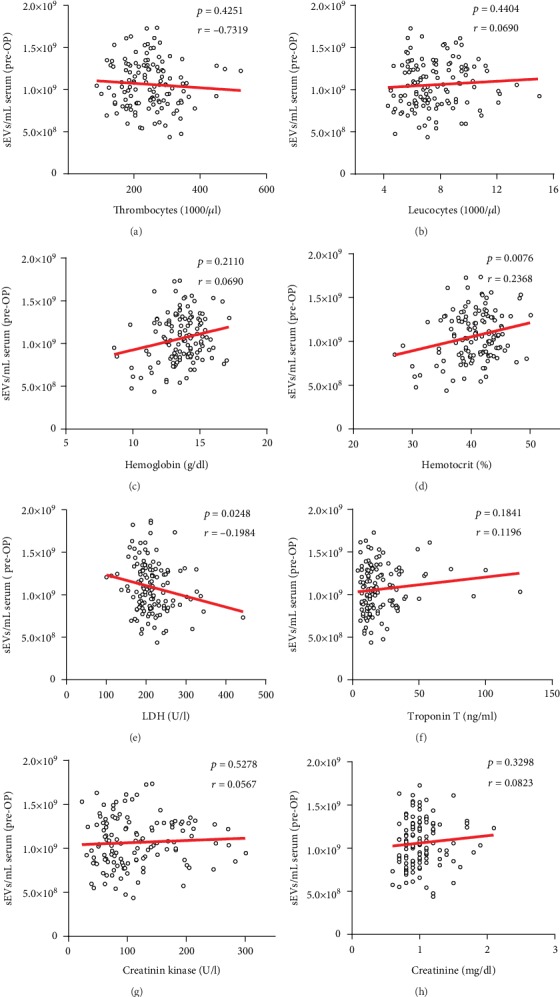
Correlation of sEVs with laboratory parameters before SAVR. Linear regression of sEVs with thrombocytes (a), leucocytes (b), hemoglobin (c), hematocrit (d), lactate dehydrogenase (e), hsTnT (f), creatinine kinase (g), and creatinine (h) in patients before undergoing SAVR (*n* = 135).

No significant correlation could be detected between the pre-OP levels of sEVs and thrombocytes (Figure 5(a), *p* = 0.4251) or leucocytes (Figure 5(b), *p* = 0.4404). However, at 7 d post-OP, the levels of sEVs increased significantly and in association with the thrombocyte levels (S4A Fig, *p* = 0.0353, *r*^2^ = 0.0355). Furthermore, there was a significant positive correlation between the levels of sEVs with hemoglobin (Figure 5(c), *p* = 0.0177, *r*^2^ = 0.0445) and hematocrit (Figure 5(d), *p* = 0.0076, *r*^2^ = 0.0561), also persisting at 7 d after SAVR (S5C-D Figs, Hb: *p* = 0.0359, *r*^2^ = 0.0342; Hct: *p* = 0.0365, *r*^2^ = 0.0339) and at the 3 mo follow up (S6C-D Figs, Hb: *p* = 0.0402, *r*^2^ = 0.0371; Hct: *p* = 0.0456, *r*^2^ = 0.0361). LDH decreased with increasing levels of sEVs at every point (pre-OP: Figure 5(e), *p* = 0.0248, *r*^2^ = 0.0393; 7 d-post-OP: Fig S5E, *p* = 0.1601; 3 mo post-OP: Fig. S6E, *p* = 0.0301, *r*^2^ = 0.0411). Further, there was no correlation between levels of sEVs and hsTnT and creatinine kinase, neither pre-OP (Figures 5(f)–(g)) nor post-OP (S5F-G Figs, S6F-G Figs). Creatinine increased with a significant association with levels of sEVs 7 d post-OP (S5H Fig, *p* = 0.0019, *r*^2^ = 0.0758), but neither at pre-OP time point (Figure 4(h), *p* = 0.5298) nor 3 mo post-OP (S6H Fig, *p* = 0.0989).

### 3.8. Circulating sEVs as Predictor for Patient-Prosthesis Mismatch and LV-Mass Regression

**Figure 6:**
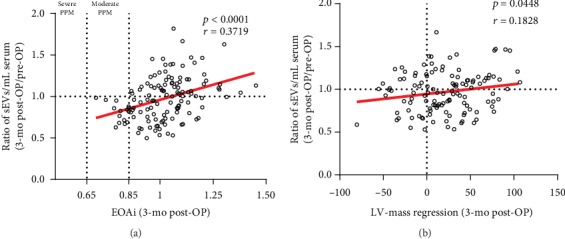
Changes of circulating sEVs as predictor for PPM and LV-mass regression. Linear regression of ratios of sEVs (3 mo post-OP/pre-OP) with emerging PPM (a) and LV-mass regression (b) in patients undergoing SAVR (*n* = 135).

A total of 15 moderate PPMs were detected at the 3 mo follow-up. There was a positive significant correlation between the 3 mo post-OP EOAi and the increase of circulating sEVs (Figure 6(a), *p* < 0.0001, *r*^2^ = 0.1383) and a slight correlation with LV-mass regression (Figure 6(b), *p* = 0.0448, *r*^2^ = 0.0334). However, increasing levels of circulating sEVs from pre-OP to 3 mo post-OP correlated significantly with a lower pre-OP BMI in both groups (S7 Fig, *p* < 0.0001, *r*^2^ = 0.1766).

## 4. Discussion

Our understanding of the biological functions of circulating vesicles has developed enormously in a short period and seems poised to expand significantly in the near future [24–26]. In the last several years, research on the biology, function, and potential application of sEVs has increased exponentially [25–27]. By now, because of technical difficulties regarding the analysis of small circulating vesicles (<1 *μ*m), a large part of published work in this area is mainly focused on larger MPs (600 nm-1 *μ*m) while disregarding sEVs [28–30]. To our best knowledge, this is the first study systematically applying NTA to examine the course of circulating sEVs in patients undergoing SAVR for AVS. Here, we performed NTA preoperatively and at three follow-up points and correlated these values with echocardiographic characteristics and blood parameters to gain further insight into the evolution of sEVs along SAVR treatment. Our data indicate that in patients with AVS, circulating sEVs may be altered and their course may be associated with some aspects of the clinical course. Therefore, it may be speculated that sEVs may take part in mediating cell-cell communication, which appears not to be affected by disease severity and may play an active role in the adaptive response of the body after SAVR. The herein presented data suggest that AVS does not promote the release of sEVs and that, in contrast to larger MPs, shear stress is not a trigger for the formation and secretion of these nano-sized vesicles. Further, we pointed out a correlation between circulating sEVs and erythrocytes as well as LDH and creatinine levels in peripheral blood. Analysis of circulating sEVs could have a prognostic value to estimate emerging PPMs and adverse outcomes in patients undergoing SAVR.

Laminar shear stress, a mechanical force generated by blood flow, is known to have major impact on the formation and release of MPs. It is described that blood shear stress caused by AVS leads to the generation of platelet MPs which then contribute either directly or indirectly via activation of endothelial cells, which is reflected by the release of endothelial MPs and by activation of monocytes to further impairment of AV function and progression of CAVD [17]. In contrast to these larger MPs, our findings demonstrate that the release of sEVs does not correlate with high transvalvular gradients and is not triggered by shear stress. The extent of sEVs release is rather regulated by different cellular conditions, such as intracellular calcium changes and potassium-induced cell depolarization or by external factors such as reactive oxygen species and inflammatory stimuli [31]. In our study, there was no correlation between the levels of circulating sEVs and respective LV-mass, LV-mass index, or RWT, which suggests that the hypertrophic responses of the LV may not be directly related to the secretion of sEVs.

In the past decade, there was an extraordinary explosion of research in the field of sEVs. Circulating sEVs have gone from being considered as useless cellular metabolic waste disposal to play an important part in mediation of cell-to-cell communication [32, 33]. In our study, as early as 7 days after SAVR, there was a marked increase of circulating sEVs before returning to initial values after 3 mo. The higher levels of circulating sEVs 7 days after SAVR could be related to the general response of the body and the physical recovery following SAVR. From this, one can infer that sEVs-mediated cell-cell communication may play a role in the recovery after major surgical interventions. Moreover, the normalized values of circulating sEVs 3 mo after SAVR indicate that sEVs, in contrast to large MPs, may be not generated as a response to the pathological progression of AVS. Rather, circulating sEVs may provide a permanent communication system, which is quickly regenerated after a certain event here, i.e., major surgical interventions. Recent studies confirmed that sEVs, which deliver specific cargoes to the recipient cells, orchestrate the regeneration process in various pathological settings by improving the microenvironment to promote cell survival, controlling inflammation, repairing injury, and enhancing the healing process [34]. Further, cardiac sEVs are believed to trigger the release of progenitor cells and to initiate myocardial repair [35]. Overall, the so far described role of circulating sEVs in processes that greatly affect tissue regeneration suggests a considerable therapeutic potential in the context of regenerative medicine.

In the present study, levels of circulating sEVs increased with higher BMI, while there was no correlation with age or gender. A possible explanation is the higher quantity of adipose tissue, where sEVs are linked to lipid metabolism and obesity-related insulin resistance and sEVs secreted by adipose tissue-derived stem cells are involved in angiogenesis, immunomodulation, and tumor development [36].

It is described that particular red blood cells (RBCs) are able to generate a great variety of circulating vesicles, including both large MPs and sEVs, which then may translocate to almost all tissues in the body without being hindered by any biological barrier [37]. Further, RBC-derived sEVs are capable of stimulating peripheral blood mononuclear cells (PBMCs) and provoking immune response by triggering proinflammatory cytokine secretion [38]. In our study, there was a correlation between circulating sEVs and respective hematocrit and hemoglobin values, both before SAVR as well as at the two analyzed follow-up points. These findings indicate that erythrocytes could possibly be one of the main sources of circulating sEVs and that erythrocyte-derived sEVs may have an active function in mediating cell-cell communication within blood cells and to peripheral tissues.

Further, laboratory parameters such as serum LDH and creatinine levels correlated with circulating sEVs. While there was no correlation with hsTnT and creatinine kinase, there was a correlation of lower levels of sEVs with higher LDH levels. Further, 7 d after SAVR creatinine levels correlated with sEVs. In general, LDH is released into the blood following cell injury or necrosis. Hence, in the clinical setting, LDH is used as a surrogate marker for tissue injury and may also be used as a marker for hemolysis [39]. The serum creatinine level reflects the balance of constant production by muscle tissue on the one hand and renal clearance on the other hand, and it serves as an important indicator of renal function [40]. In front of this background, we interpret the findings of this study in the sense that higher levels of circulating sEVs may be an indicator for favorable recovery after SAVR.

One or perhaps the most important biological use of circulating sEVs is their potential application as biomarkers in clinical diagnostics [41]. Most of the current studies in this field mainly focus on discovering exosomal biomarkers for early detection and prediction of prognosis in the field of oncology [42]. However, sEVs remain largely unexplored for clinical use in the field of cardiovascular medicine. In our study, we evaluated the use of circulating sEVs as potential biomarkers for emerging PPMs and LV-mass regression after SAVR. Interestingly, all patients with a moderate PPM 3 mo after SAVR had lower levels of circulating sEVs compared to their respective status before surgery. Further, in the same way, patients with impaired or absent LVM regression or even an increase of the LVM tend to have lower levels of circulating sEVs after SAVR compared to respective preoperative values: a finding that fits to the other aforementioned observations. In general, patients with higher BMI have a higher risk for emerging PPMs. In our study, patients with higher BMI tend to have lower levels of circulating sEVs after SAVR compared to preoperative values. Unfortunately, based on our limited numbers of patients with moderate PPMs, we cannot clearly state that circulating sEVs are a reliable marker for an emerging PPM or absent LV-mass regression. However, the presented results suggest that lower levels of circulating sEVs may be an indicator for negative ramifications after SAVR.

One limitation of our study is that our sample size with 135 patients may be not big enough yet, particularly with respect to the analysis of subcohorts, e.g., patients with postoperative PPM. Not all patients receiving AVR and fitting the inclusion criteria could be enrolled in our study and 69 patients had to be excluded due to intraoperative change of strategy with varying additional surgical procedures (e.g., replacement of the ascending aorta, use of a mechanical prosthesis) or due to missing follow-up blood samples from patients with a remote residence who were followed-up by phone. Another limitation of the present study is the general problem with the analysis of sEVs and the technical approach for isolation and analysis. In preparation for this study, we tested different isolation techniques as well as validated and standardized the analysis method. We deliberately choose to use a precipitation reagent, which, in contrast to purely ultracentrifugation-based protocols, results in a complete precipitation of virtually all sEVs and yields reproducible results, as confirmed by multiple isolation and analysis of the same sample (data not shown). And yet, a universal methodological approach isolation and analysis of sEVs is currently missing.

## 5. Conclusions

To the best of our knowledge, this is the first study analyzing the course of sEVs in a prospective longitudinal study on patients with AVS undergoing SAVR. Circulating sEVs may take an important part in mediating cell-cell communication in patients with AVS. Further, lower levels of sEVs associated with less favorable echocardiographic and laboratory parameters after three months, thus possibly representing an indicator for adverse outcome after SAVR for AVS.

## Figures and Tables

**Table 1:** Patients' characteristics of the study cohort before SAVR.

	Mean ± SD or *n* (%)
*n*	135
Age (years)	73.3 ± 7.05
Male	82 (61)
Weight (kg)	81.2 ± 16.9
BMI	27.9 ± 4.67
NYHA classification	
I	18 (13)
II	43 (32)
III	67 (50)
IV	7 (5)
Cardiac decompensation	10 (7.4)
Syncopation	13 (10)
Hemorrhage	2 (1.5)
HTN	110 (81)
Pulmonary hypertension	21 (16)
Diabetes mellitus type 2	34 (25)
Cardiac arrhythmia	28 (21)
Dyslipidemia	58 (43)
Liver disease	4 (2.9)
Lung disease	19 (14)
GFR	
<30 mL/min	1 (1)
30–60 mL/min	36 (27)
>60 mL/min	98 (72)
Bicuspid aortic valve	18 (13)
ES II	2.14 ± 1.41
STS score	1.66 ± 0.69

BMI: body mass index; NYHA: New York Heart Association; CAD: coronary artery disease; HTN: hypertension; GFR: glomerular filtration rate; ES: Euroscore; STS: Society of Thoracic Surgeons.

**Table 2:** Echocardiographic parameters before SAVR, after 7 days and at 3 mo follow-up.

	Pre-SAVR	7 days		3 months	
	Mean ± SD	Mean	*p* value	Mean ± SD	*p* value
EF (%)	58.4 ± 7.26	58.4 ± 5.75	0.9947	60.1 ± 5.92	0.0719
LVEDd (mm)	48.3 ± 4.11	47.6 ± 3.88	0.2511	48.5 ± 3.67	0.9451
LVESd (mm)	33.1 ± 5.85	33.1 ± 4.66	0.9938	31.3 ± 5.37	0.0194
IVSd (mm)	13.8 ± 1.99	13.2 ± 2.22	0.0374	12.7 ± 1.52	<0.0001
PWd (mm)	11.6 ± 1.44	11.1 ± 1.63	0.0049	10.6 ± 1.23	<0.0001
AWd (mm)	11.0 ± 1.15	10.6 ± 1.37	0.0177	10.4 ± 1.05	<0.0001
Peak gradient (mmHg)	65.4 ± 19.7	17.7 ± 6.15	<0.0001	17.4 ± 5.87	<0.0001
Mean gradient (mmHg)	39.1 ± 12.8	10.2 ± 3.49	<0.0001	9.15 ± 3.08	<0.0001
Peak aortic jet velocity (m/s)	3.99 ± 0.61	2.07 ± 0.35	<0.0001	2.05 ± 0.34	<0.0001
EOA (cm^2^)	0.76 ± 0.16	1.96 ± 0.26	<0.0001	1.93 ± 0.22	<0.0001
Indexed EOA (cm^2^/m^2^ BSA)	0.41 ± 0.08	1.02 ± 0.15	<0.0001	1.02 ± 0.14	<0.0001
Shear stress (Vmax/LV-EF)	0.07 ± 0.01	0.03 ± 0.01	<0.0001	0.03 ± 0.01	<0.0001
Calculated LV-mass (g)	233 ± 48.2	214 ± 48.5	0.0027	212 ± 44.5	0.0006
LV-mass index (g/m^2^)	121 ± 21.9	111 ± 23.1	0.0006	111 ± 19.1	0.0002
RWT	0.46 ± 0.06	0.45 ± 0.07	0.3011	0.43 ± 0.05	0.0003

SAVR: surgical aortic valve replacement; EF: ejection fraction; LVEDD: left ventricular end-diastolic diameter; LVESd: left ventricular end-systolic diameter; IVSd: intraventricular septal end-diastolic diameter; PWd: posterior wall diameters; AWd: anterior wall diameter; EOA: effective orifice area; BSA: body surface area; Vmax: peak aortic jet velocity; LV: left ventricular; RWT: relative wall thickness. Significances are expressed vs. baseline parameters before SAVR.

**Table 3:** Laboratory parameters before SAVR, after 7 days and at 3 mo follow-up.

	Pre-SAVR	7 days		3 months	
	Mean ± SD	Mean ± SD	*p* value	Mean ± SD	*p* value
Thrombocytes (1000/*μ*l)	248 ± 87.1	258 ± 99.4	0.5321	240 ± 83.6	0.7085
Leucocytes (1000/*μ*l)	7.55 ± 2.11	9.77 ± 3.51	<0.0001	7.38 ± 2.02	0.8289
Hemoglobin (g/dl)	13.5 ± 1.58	10.6 ± 1.12	<0.0001	12.8 ± 1.36	0.0002
Hematocrit (%)	40.1 ± 4.19	32.4 ± 3.72	<0.0001	39.3 ± 3.69	0.0488
Creatinine (mg/dl)	1.03 ± 0.28	0.97 ± 0.31	0.2315	1.08 ± 0.32	0.3443
Creatinine kinase (U/l)	123 ± 96.9	77.3 ± 55.1	<0.0001	102 ± 69.5	0.0305
C-reactive protein (mg/l)	0.61 ± 1.84	6.47 ± 4.91	<0.0001	0.72 ± 1.37	0.9412
hsTnT (ng/ml)	20.1 ± 18.3	329 ± 613	<0.0001	21.7 ± 24.7	0.9997
LDH (U/l)	219 ± 68.2	329 ± 83.1	<0.0001	232 ± 50.6	0.2267
Urea (mg/dl)	38.4 ± 10.6	35.2 ± 17.8	0.2658	44.6 ± 22.6	0.0124
GOT (U/l)	28.5 ± 19.1	44.9 ± 54.8	0.0003	25.6 ± 9.11	0.7150

hsTnT: high sensitive Troponin T; LDH: lactate dehydrogenase (LDH); GOT: glutamate oxaloacetate transaminase.

## Data Availability

The data sets generated and/or analyzed during the current study are available from the corresponding author on reasonable request.

## Supplementary Materials

Supplementary Materials The supplementary materials include (1) an extra table with the applied acquisition parameters for nanoparticle tracking analysis, (2) one figure with size distribution curves and representative images as well as descriptive statistics of the captured sEVs for each time point of one patient, (3) one figure with the comparison of serum levels of sEV of patients receiving SAVR or without concomitant coronary artery bypass grafting, (4) two figures showing the correlation of circulating sEVs with echocardiographic parameters 7 d and 3 mo after SAVR, (5) two figures showing the correlation of circulating sEVs with laboratory parameters 7 days and 3 mo after SAVR, and (6) one figure with the correlation of sEV ratios with BMI.
