# Supplementary material for: The Course of Circulating Small Extracellular Vesicles in Patients Undergoing Surgical Aortic Valve Replacement
Source: Biomed Res Int. 2020 Apr 18;2020:6381396. doi: 10.1155/2020/6381396 (PMC7193280; doi:10.1155/2020/6381396)
Supplement: Supplementary Materials — The supplementary materials include (1) an extra table with the applied acquisition parameters for nanoparticle tracking analysis, (2) one figure with size distribution curves and representative images as well as descriptive statistics of the captured sEVs for each time point of one patient, (3) one figure with the comparison of serum levels of sEV of patients receiving SAVR or without concomitant coronary artery bypass grafting, (4) two figures showing the correlation of circulating sEVs with echocardiographic parameters 7 d and 3 mo after SAVR, (5) two figures showing the correlation of circulating sEVs with laboratory parameters 7 days and 3 mo after SAVR, and (6) one figure with the correlation of sEV ratios with BMI. [file 6381396.f1.pdf]

Supplementary information

The course of circulating small extracellular vesicles in patients undergoing surgical aortic valve replacement

Andreas Weber[1], Shining Sophie Liu[1], Letizia Cardone[1], Philipp Rellecke[1], Stephan Sixt[2], Artur Lichtenberg[1]*, Payam Akhyari[1]

[1] Department of Heart Surgery, Heinrich-Heine-University, Medical Faculty, Düsseldorf, Germany

[2] Department of Anaesthesiology, Heinrich-Heine-University, Medical Faculty, Düsseldorf, Germany

*** Corresponding author:** Artur Lichtenberg

Mailing address: Department of Cardiovascular Surgery

Heinrich-Heine-University, Medical Faculty

Moorenstraße 5

40225 Düsseldorf

Germany

phone: +49-(0)211-8118331

fax:+49-(0)211-8118333

e-mail: Artur.Lichtenberg@med.uni-duesseldorf.de

Table S1: Acquisition parameters for nanoparticle tracking analysis.

Acquisition parameters	
Sensitivity [%]	70
Shutter	70
Min. Brightness	20
Max Size [nm]	500
Min. Size [nm]	20
Polarity	Negative
Voltage	Off
Particle Drift at 0V [µm/s]	< 20
Positions	11
Cycles	10
Multiple acquisitions	3
Time Delay [min]	0

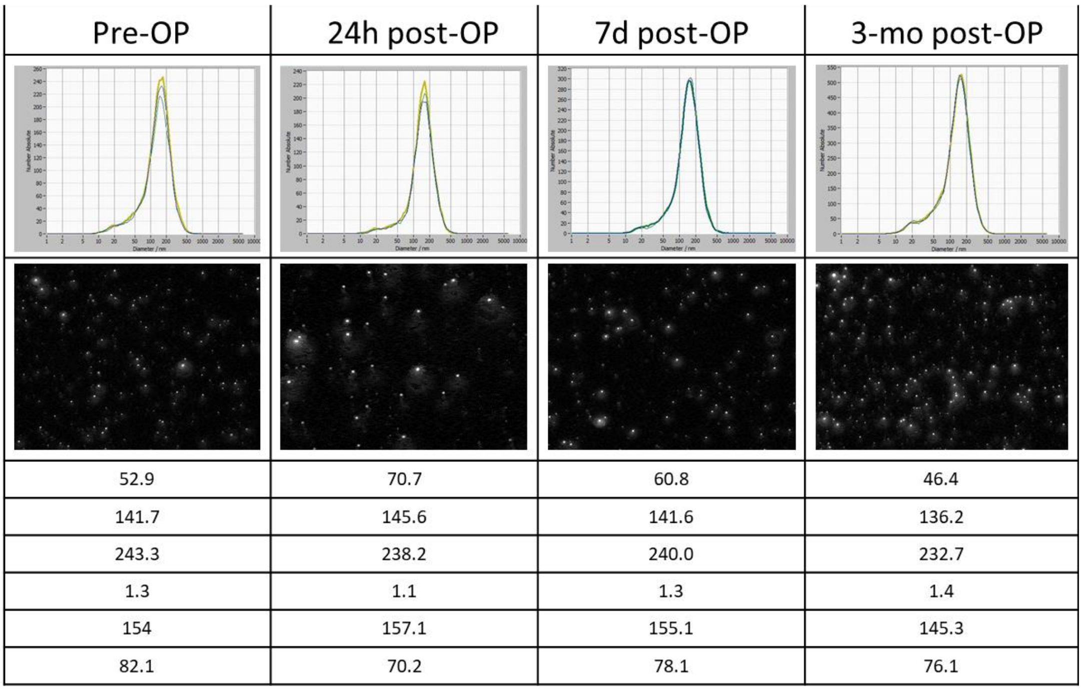

	Pre-OP	24h post-OP	7d post-OP	3-mo post-OP
X10 (nm)	52.9	70.7	60.8	46.4
X50 (nm)	141.7	145.6	141.6	136.2
X90 (nm)	243.3	238.2	240.0	232.7
Span	1.3	1.1	1.3	1.4
Mean (nm)	154	157.1	155.1	145.3
StdDev (nm)	82.1	70.2	78.1	76.1

S1 Fig.: NTA analysis of sEVs. Size distribution curves and representative images as well as descriptive statistics (means of all patients) of the captured sEVs for each time point of one patient.

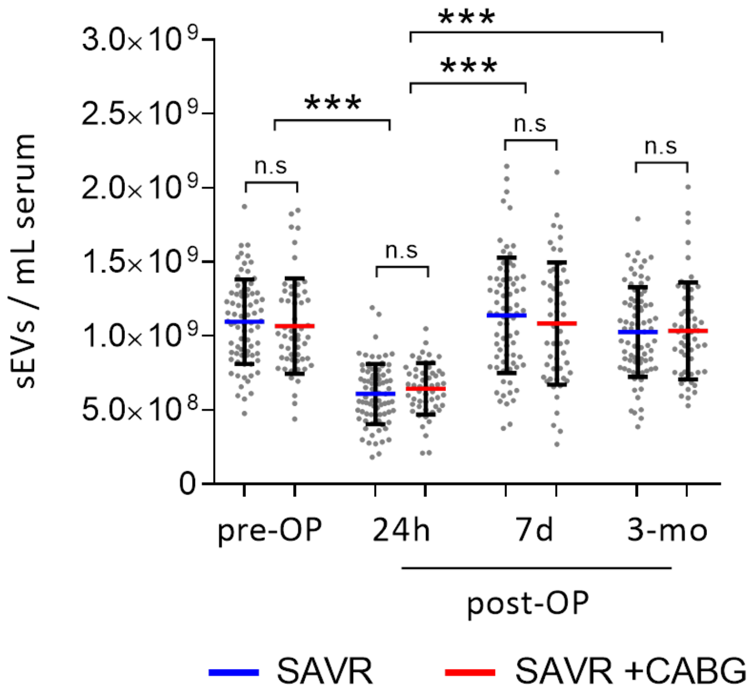

S2 Fig.: Serum levels of circulating sEVs. Comparison of sEVs of patients receiving SAVR with (red line) or without concomitant coronary artery bypass grafting (blue line) measured at four time points. pre-OP, sample from the day prior to operation; 24h post-OP, sample from the first postoperative day; 7d post-OP, sample taken on 7th postoperative day; and 3-mo post-OP, sample taken on the occasion of follow-up analysis three months after surgery. Mean ± SD; *** $p < 0.001$.

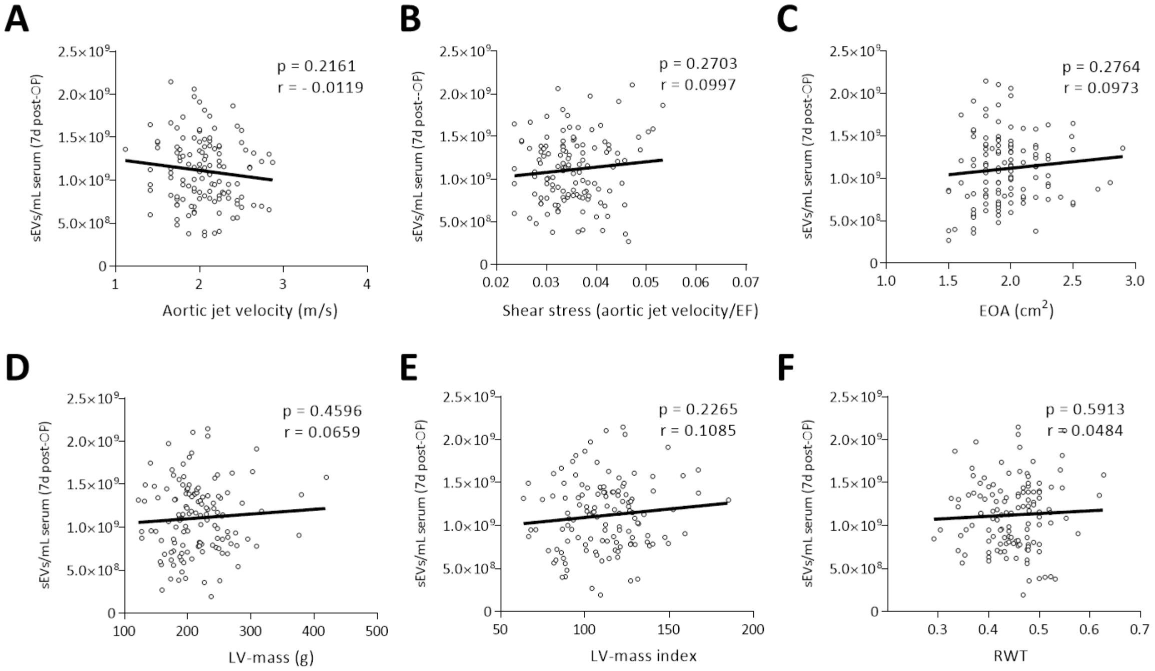

S3 Fig.: Correlation of circulating sEVs with echocardiographic parameters 7d after SAVR.

Linear regression of sEVs with aortic jet velocity (A), shear stress at the level of the aortic valve (B), effective orifice area (C), LV-mass (D), LV-mass index (E) and relative wall thickness (F) 7d after SAVR.

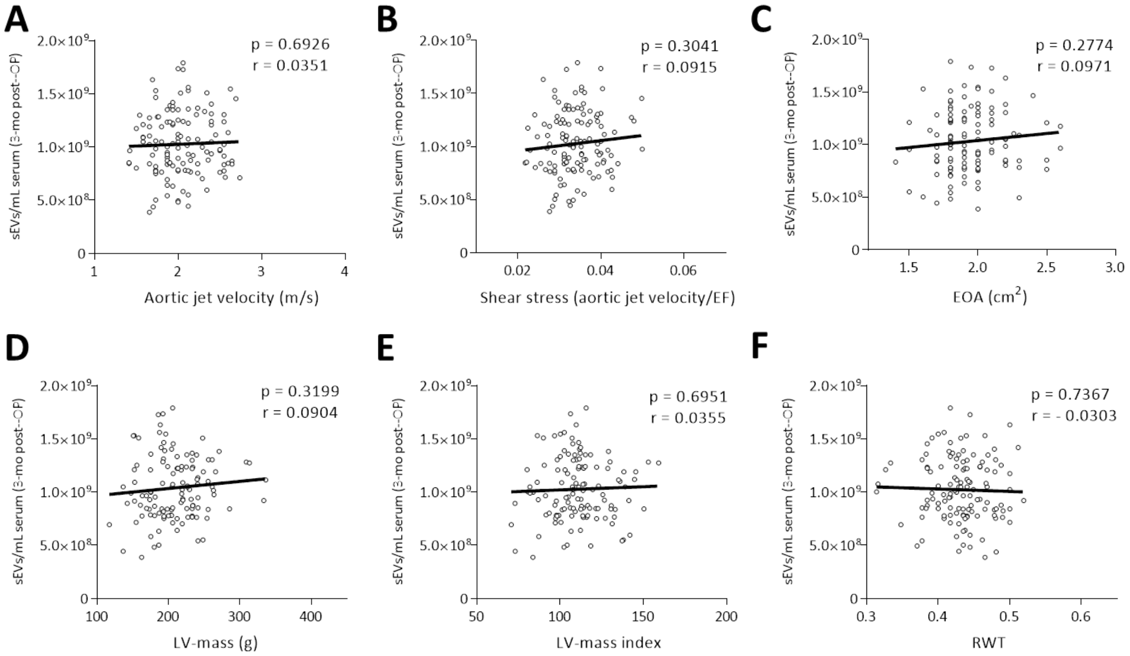

S4 Fig.: Correlation of circulating sEVs with echocardiographic parameters at follow-up three months after SAVR. Linear regression of sEVs with aortic jet velocity (A), shear stress at the level of the aortic valve (B), effective orifice area (C), LV-mass (D), LV-mass index (E) and relative wall thickness (F) 3-mo after SAVR.

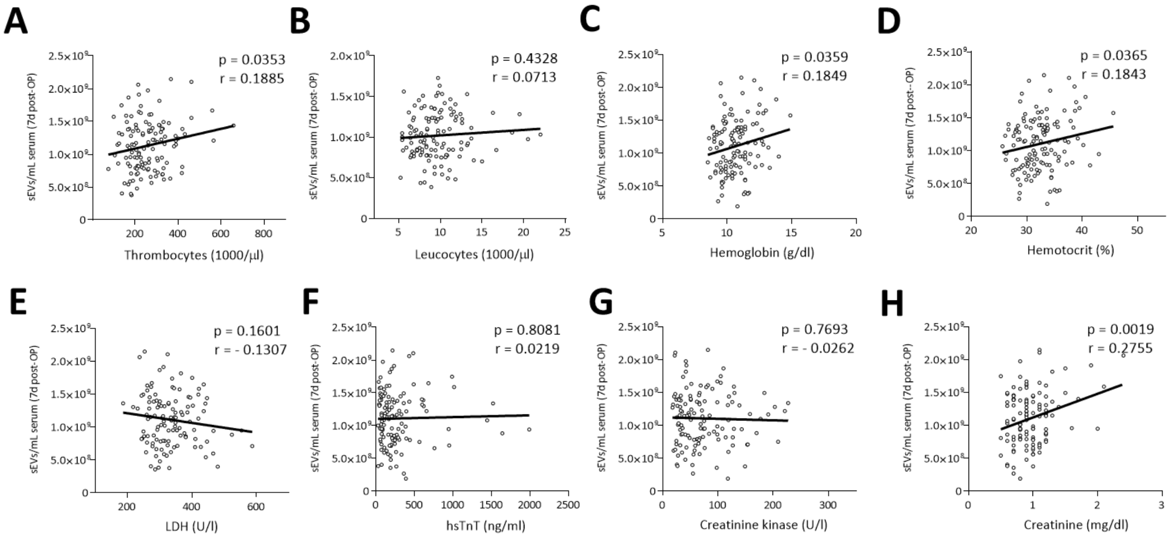

S5 Fig.: Correlation of circulating sEVs with laboratory parameters 7 days after SAVR. Linear regression of sEVs with thrombocytes (A), leucocytes (B), hemoglobin (C), hematocrit (D), lactate dehydrogenase (E), high sensitive troponin T (F), creatinine kinase (G) and creatinine (H) 7 d after SAVR.

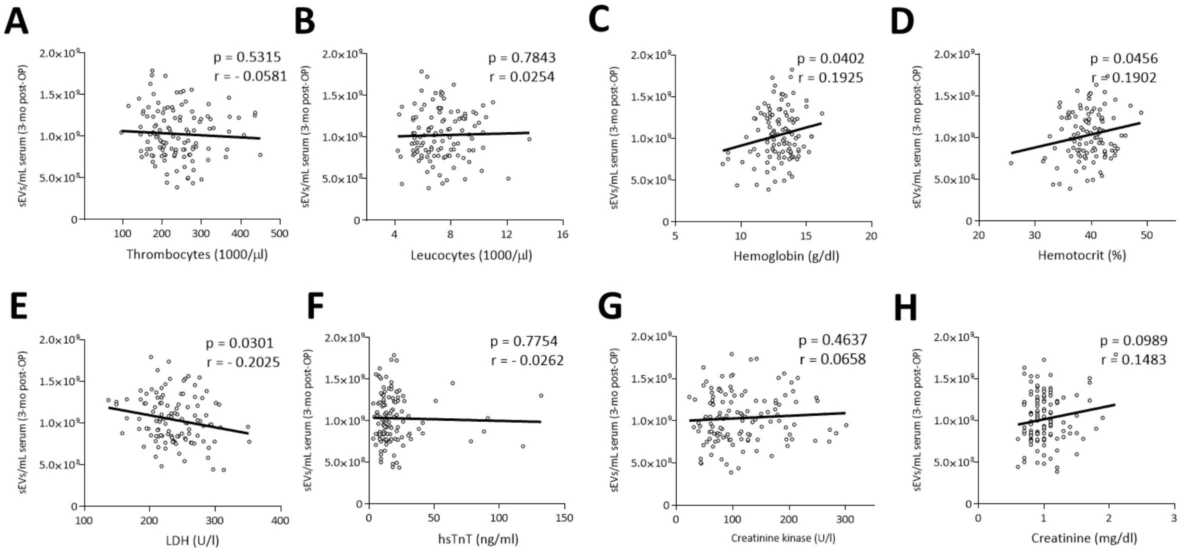

S6 Fig.: Correlation of circulating sEVs levels with laboratory parameters at follow-up three months after SAVR. Linear regression of sEVs with thrombocytes (A), leucocytes (B), haemoglobin (C), haematocrit (D), lactate dehydrogenase (E), high sensitive troponin T (F), creatinine kinase (G) and creatinine (H) 3-mo after SAVR.

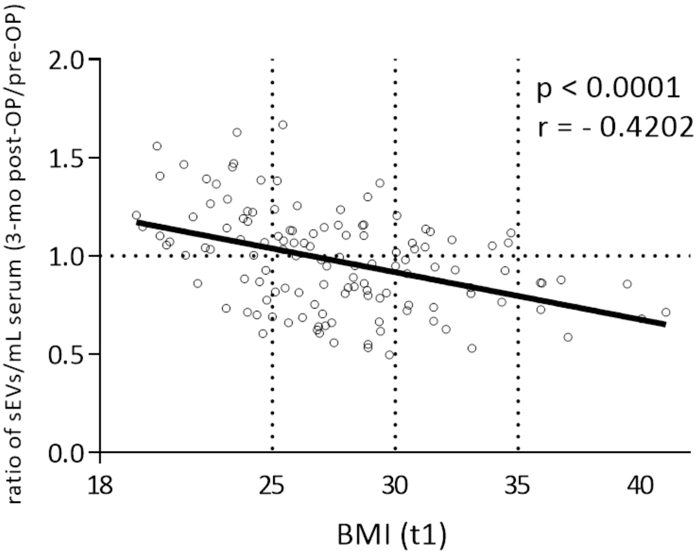

S7 Fig.: Correlation of sEV ratios with BMI. Linear regression of sEV ratios with BMI in patients undergoing SAVR. Ratio of sEVs represents the levels of circulating sEVs at 3-mo post-OP divided by the respective pre-OP value. BMI, body mass index.
